# Hypoglycemic Properties of Oxovanadium (IV) Coordination Compounds with Carboxymethyl-Carrageenan and Carboxymethyl-Chitosan in Alloxan-Induced Diabetic Mice

**DOI:** 10.1155/2011/691067

**Published:** 2011-07-26

**Authors:** Hongyu Zhang, Yuetao Yi, Dawei Feng, Yipeng Wang, Song Qin

**Affiliations:** ^1^Yantai Institute of Coastal Zone Research, Chinese Academy of Sciences, Yantai 264003, China; ^2^Graduate School of Chinese Academy of Sciences, Beijing 100049, China; ^3^Biological Resources Laboratory, Yantai Institute of Coastal Zone Research, Chinese Academy of Sciences, Yantai, 264003, China

## Abstract

In order to avoid low absorption, incorporation, and undesirable side effects of inorganic oxovanadium compounds, the antidiabetic activities of organic oxovanadium (IV) compounds in alloxan-induced diabetic mice were investigated. Vanadyl carboxymethyl carrageenan (VOCCA) and vanadyl carboxymethyl chitosan (VOCCH) were synthesized and administrated through intragastric administration in different doses for 20 days in alloxan-induced diabetic mice. Glibenclamide was administrated as the positive control. Our results showed that low-dose group, middle-dose group, and high-dose group of VOCCA and VOCCH could significantly reduce the levels of blood glucose (*P* < 0.05) compared with untreated group, but not in normal mice. Besides, high-dose groups of VOCCA and VOCCH exhibited more significant hypoglycemic activities (*P* < 0.01). After treated with VOCCH, the oral glucose tolerance of high-dose group of VOCCH was improved compared with model control group (*P* < 0.05).

## 1. Introduction

Diabetes mellitus (DM), which results from insulin deficiency or insulin resistance, is a serious chronic metabolic disease [1]. The DM includes insulin-dependent type 1 DM and non-insulin-dependent type 2 DM. Until now, type 1 DM can only be controlled by subcutaneous injections of insulin, which causes many problems to the patients. In order to treat type 2 DM, some drugs have also been synthesized [2], for example, sulfonylureas, sulfonamides, biguanides, thiozolidinediones, and so on. Orally active therapeutic agents instead of painful insulin injections for type 1 DM and synthetic drugs without side effects for type 2 DM have become an urgent and important requirement.

Vanadium is not only an important trace element for organisms, but also the necessary element for human body [3]. It has been demonstrated that many vanadium compounds possess therapeutic effects as insulin mimetics [4, 5]. Heyliger et al. first reported the insulin mimetic activity of oral vanadate *in vivo* in 1985 [6]. Since then, extensive studies have been carried out to explore vanadium chemistry, including the synthesis of novel complexes and their antidiabetic activities both *in vitro* and *in vivo* [7–16]. Furthermore, many clinical trials of vanadium compounds have also been reported [17–20], in which vanadium salts such as VOSO_4_ and NaVO_3_ were administered to diabetic patients. In order to enhance both lipophilicity and bioavailability of vanadium compound, overcome the disadvantage of side effects, increase the half-life of the compound, decrease systemic drug toxicity, improve treatment absorption rates, and provide protection for pharmaceuticals against biochemical degradation, two types of less toxic vanadyl (+4 oxidation state of vanadium) complexes with different coordination structures were synthesized and examined.

In addition, the polysaccharide polymer is biodegradable and biocompatible and would be effective in enhancing drug bioavailability through the mechanism of delaying release. In order to investigate the impact of different organic ligands on vanadyl complexes, vanadyl carboxymethyl k-carrageenin and vanadyl carboxymethyl chitosan were synthesized. The present study was performed to investigate the antidiabetic properties of these two new vanadyl complexes in alloxan-induced diabetic mice.

## 2. Materials and Methods

### 2.1. Materials and Equipments

K-carrageenin and chitosan with a deacetylation degree of 95.3% were purchased from Qingdao Baicheng Biochemical Corp (China), their viscosity-average molecular weights were 3.77 × 10^5^ and 2.0 × 10^5^, respectively. Isopropanol, glucose, alloxan, and vanadyl sulfate hydrate (VOSO_4_·*x*H_2_O, *x* = 3 to 5) were purchased from the Sigma-Aldrich Chemical Co. Glibenclamide was purchased from Pacific pharmaceutical technology group. Ethanol, sodium hydroxide, and other reagents were purchased from Sinopharm Co. Kunming mice were provided by experimental animal center, Kunming. The IR spectra were measured on a JASCO-4100FT-IR spectrometer with KBr disks. The content of vanadium is measured on 7AS-986 (G) Atomic Absorption Spectrometer. Chemicals and solvents were reagent grade.

### 2.2. Synthesis of VOCCA and VOCCH

K-carrageenin (chitosan) (50 g) was added into 750 mL isopropanol and stirred for 30 min. 40 mL sodium hydroxide solution (mass fraction is 50%) was slowly dropped into the mixture (25 min) and stirred for 3 h. Then 60 g monochloroacetic acid was added into the mixture for 5 times in 30 min, and the system temperature was kept at 60°C for 4 h. After incubating for 4 h, the temperature was lowered to 25°C and the pH was adjusted to 7.0. The mixture was then concentrated with ethanol three times in volume of the mother liquid. After filtration, washing, drying, and smashing, the carboxymethyl carrageenin and carboxymethyl chitosan were obtained. 

Ashing method [21] was applied to calculate the SD of carboxymethyl groups of carboxymethyl carrageenin and carboxymethyl chitosan. Carboxymethyl carrageenin and carboxymethyl chitosan were vacuum dried at 60°C to constant mass; carboxymethyl carrageenin and carboxymethyl chitosan were heated and scorched at 700°C for 15–20 min. The residues were Na_2_O, leached with 50 mL HCl solution (0.1 moI/L), heated, and followed by residual titration with 0.1 mol/L standard NaOH. The SD of carboxymethyl groups of carboxymethyl carrageenin and carboxymethyl chitosan were calculated as the formula discussed by Wang and Ye.

Vanadyl carboxymethyl carrageenin (chitosan) (the SD of carboxymethyl carrageenin and carboxymethyl chitosan were 47.3% and 84.9%, resp.) was prepared by mixing various amounts of VOSO_4_ with carboxymethyl carrageenin (the mass ratio of V to carboxymethyl carrageenin (chitosan) was 1%) solutions under magnetic stirring for 24 h at room temperature. Ethanol four times in volume of the mother liquid was added to the solution to complete precipitation of vanadyl carboxymethyl carrageenin (chitosan) complex. The resulting precipitate was washed with ethanol and dried under a vacuum condition at room temperature, and white solid vanadyl carboxymethyl carrageenin and carboxymethyl chitosan were obtained. VOCCA and VOCCH were dissolved in tap water to the concentration of 5% (w/v) every day and kept at 4°C.

### 2.3. Vanadium Content Determination

7AS-986 (G) Atomic Absorption Spectrometry was applied to determine the content of vanadium of the complex according to the standard method of the instruction.

### 2.4. Maximal Tolerant Dose (MTD) Determination of VOCCA

The result of preliminary acute toxicity test showed no death of mouse. Afterwards, the Maximal Tolerant Dose (MTD) test was carried out. Twenty normal mice weighing 20 ± 2 g were fasted for 24 h, then VOCCA (5%, 0.8 mL) was administered intragastrically three times a day for two weeks. After two weeks, the maximum tolerated dose was calculated according to the formula:

(1) $$\text{MTD}=\frac{\text{the dose of intragastric administration}\times 3}{\text{body weight of rat}}.$$

### 2.5. Construction of Diabetic Mouse Model

Male mice weighing 20–24 g were used for animal model construction. Mice were fasted for 24 h and then intraperitoneally injected alloxan (160 mg/kg). Five days after alloxan injection, the mice were fasted for 6 h and blood samples were obtained from tail vein of the mice. The blood glucose levels were measured by the glucose oxidase method. The alloxan-injected mice with the blood glucose level between 10 and 20 mmol/L were considered as diabetic mice.

### 2.6. Glucose-Lowering Test

Normal mice and diabetic mice were randomly divided into 6 groups (10 mice per group). Normal control group: normal mice; model control group: diabetic mice treated with tap water; glibenclamide control group: diabetic mice treated with 0.2 g/kg glibenclamide; low-dose group: diabetic mice treated with 0.3125 g/kg VOCCH and 0.6250 g/kg VOCCA; middle-dose group: diabetic mice treated with 0.6250 g/kg VOCCH and 1.250 g/kg VOCCA; high-dose group: diabetic mice treated with 1.2500 g/kg VOCCH and 2.500 g/kg VOCCA. The samples above were administered intragastrically for 20 days.

### 2.7. Oral Glucose Tolerance Test (OGTT) of VOCCH

After the VOCCH was administered intragastrically in both normal and alloxan-induced diabetic mice for 20 days, oral glucose tolerance test was executed. Glucose loaded with the single dose of 2.5 g/kg was intragastrically administered to mice fasted for 6 h, and the blood glucose level was checked at 0, 0.5, 2 h.

### 2.8. Blood Sample Collection

The blood samples were obtained from tail vein of mice. Firstly, the tail of mouse was put into hot water (50°C) for several minutes and cleaned, then the tail tip was cut for 1-2 mm long and blood sample was obtained.

### 2.9. Statistical Analysis

All data were expressed as the mean ± SD. Statistical analysis was performed by one-way analysis of variance (ANOVA) followed by the least significant difference (LSD) test for the multiple comparisons among the groups. Values for *P* < 0.05 were considered statistically significant.

## 3. Result

### 3.1. Character of VOCCA and VOCCH

**Figure 1:**
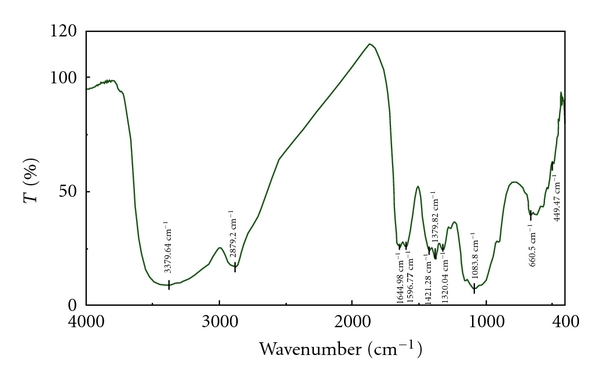
FT-IR spectra of chitosan.

**Figure 2:**
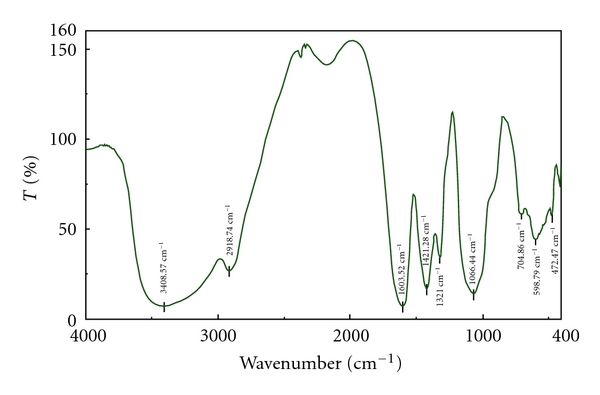
FT-IR spectra of carboxymethyl chitosan.

**Figure 3:**
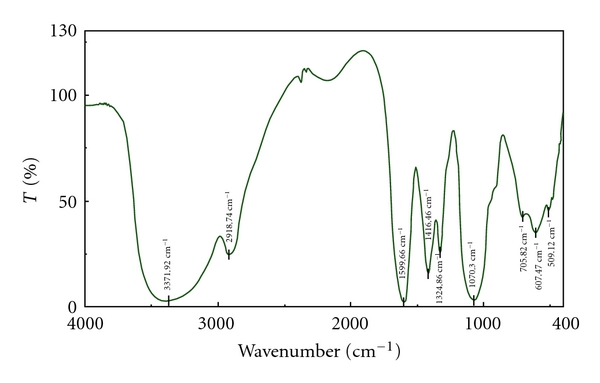
FT-IR spectra of vanadyl carboxymethyl chitosan.

Figures 1, 2, and 3 are infrared spectra of chitosan, carboxymethyl chitosan, and vanadyl carboxymethyl chitosan. Infrared spectrum (Figure 2) showed that C–OH stretching vibration absorption peak of carboxymethyl chitosan was located at 1066.44 cm^−1^, N–H and O–H at 3408.57 cm^−1^, –OH antisymmetrical stretching vibration absorption peak (COO) was located at 1603.52 cm^−1^, and its symmetrical stretching vibration (COO) absorption peak was at 1421.28 cm^−1^. 

Infrared spectrum of VOCCH (Figure 3) showed that C–OH absorption peak was displaced to 1070.30 cm^−1^, N–H and O–H absorption peaks were displaced to 3371.92 cm^−1^, OH antisymmetrical stretching vibration absorption peak and its symmetrical stretching vibration (COO) absorption peak were displaced to 1599.66 cm^−1^ and 1416.46 cm^−1^. This indicated that carboxyl, amino-group, and hydroxyl participated in the interaction of carboxymethyl chitosan and VO^2+^. The content of V in VOCCH was 0.36%.

**Figure 4:**
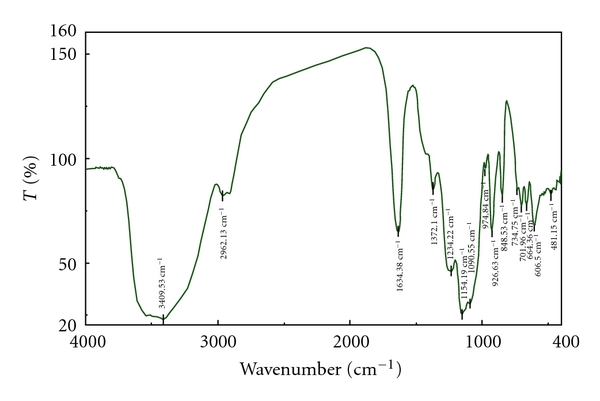
FT-IR spectra of carrageenan.

**Figure 5:**
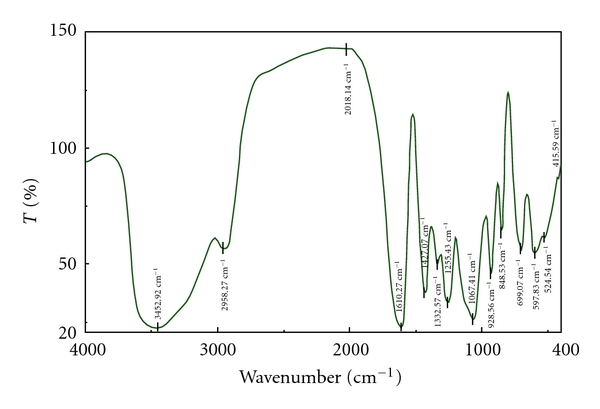
FT-IR spectra of carboxymethyl carrageenan.

**Figure 6:**
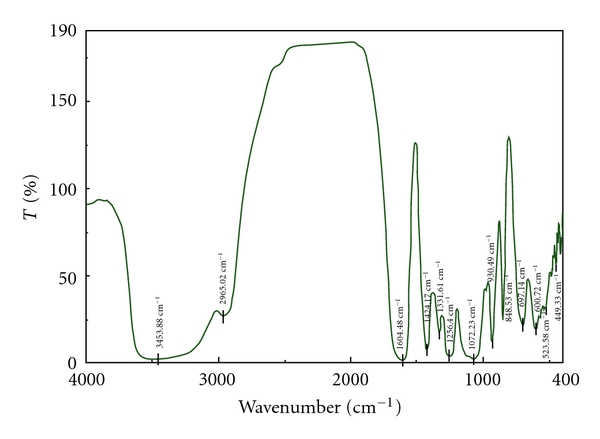
FT-IR spectra of vanadyl carboxymethyl carrageenan.

Figures 4, 5, and 6 are the IR spectra of carrageenan, carboxymethyl carrageenan, and vanadyl carboxymethyl carrageenan. Compared to the carrageenan, carboxymethyl carrageenan showed characteristic peak 1610 cm^−1^ of C=O group. Further, the peak of C=O in carboxymethyl carrageenan at 1610 cm^−1^ shift to 1604 cm^−1^ of vanadyl carboxymethyl carrageenan, also the shifts of absorption peaks of S=O and OH were observed (Figure 6). These results clearly indicated the incorporation of carboxymethyl group and VO^2+^ into carrageenan and carboxymethyl carrageenan, respectively. The content of V of VOCCA measurement on Atomic Absorption Spectrometry was 0.18%.

### 3.2. MTD of VOCCA

In the test of maximal tolerant dose, there were no convulsions, vomiting, or other negative symptoms.

(2) $$\text{MTD}=0.8\;\text{mL}\times 5\;\text{g}/\text{mL}\times \frac{3}{20}\;\text{g}=6.0\;\text{g}/\text{Kg}.$$

### 3.3. Glucose-Lowering Studies and OGTT

**Figure 7:**
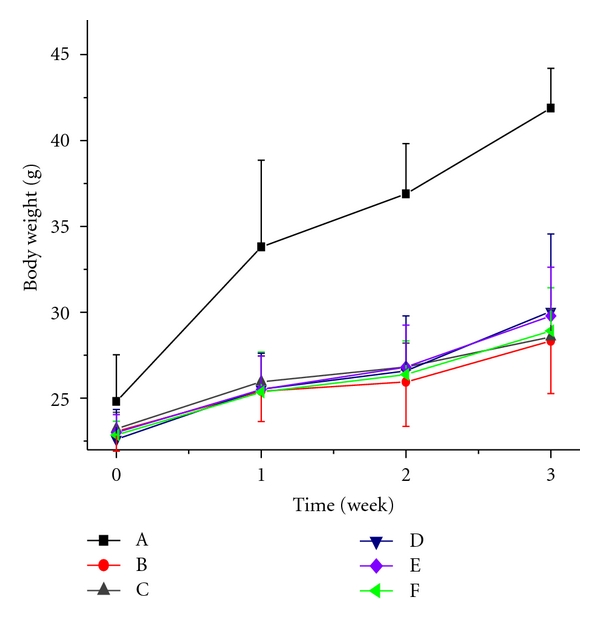
Impacts of intragastric VOCCA administration on body weight in both normal and alloxan-diabetic mice; (A): normal control group, (B): model control group, (C): glibenclamide control group, (D): low dose group, (E): middle dose group, (F): high dose group.

**Figure 8:**
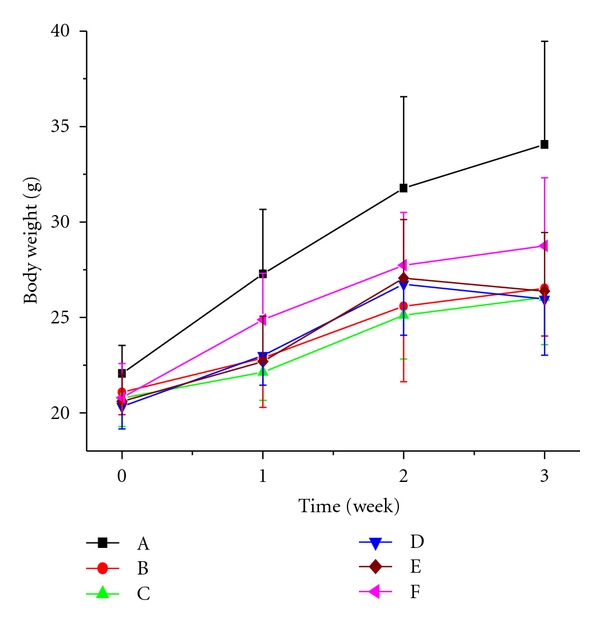
Impacts of intragastric VOCCH administration on body weight in both normal and alloxan-diabetic mice; (A): normal control group, (B): model control group, (C): glibenclamide control group, (D): low-dose group, (E): middle-dose group, (F): high-dose group.

In Figures 7 and 8, the body weights of each group before and after treatment with VOCCA and VOCCH are shown. In this study, the body weights of diabetic mice were lowered compared with those of normal control mice throughout the experimental period of 20 days. At the end of the experiment, the body weights of vanadium-treated groups exhibited no significant deviation with model control group and glibenclamide control group.

The body weight profile indicated that there was no negative impact on body weight of the mice after treated with both VOCCA and VOCCH. These complexes showed comparable results with other vanadyl coordination compounds [22].

**Figure 9:**
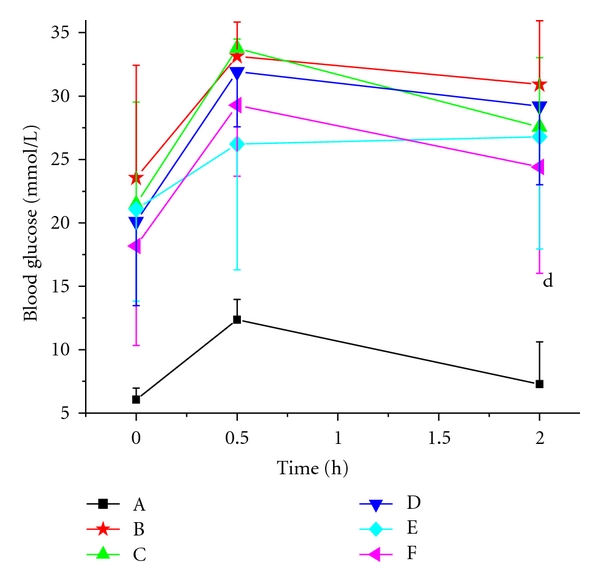
Impacts of VOCCH on glucose tolerance in normal mice and alloxan-diabetic mice. (A): normal control group, (B): model control group, (C): glibenclamide control group, (D): low dose group, (E): middle dose group, (F): high dose group. *d* ^*P*^ < 0.05, versus model control group.

The changes of blood glucose levels are shown in Tables 1 and 2. The initial blood glucose levels of the diabetic mice were similar. Low-dose group, middle-dose group, and high-dose group of VOCCA could statistically significantly reduce the levels of blood glucose (*P* < 0.05) compared with model control group, and high-dose group of VOCCA had more significant hypoglycemic activity (*P* < 0.01) (Table 1). There was no obvious difference among low-dose group, middle-dose group, and high-dose group compared with glibenclamide control group (*P* > 0.05). Low-dose group, middle-dose group, and high-dose group of VOCCH could statistically significantly reduce the levels of blood glucose (*P* < 0.05) compared to model control group. However, high-dose group showed more apparent effect than glibenclamide control group (*P* < 0.01) (Table 2). Furthermore, the oral glucose tolerance was improved in diabetic animals after treated with VOCCH (*P* < 0.05) (Figure 9). Meanwhile, low-dose group, middle-dose group, and high-dose group of VOCCA and VOCCH showed no negative influence on blood glucose levels of normal mice (Tables 1 and 2). 

Among low-dose group, middle-dose group and high-dose group of VOCCA and VOCCH at the same concentration of vanadium, the blood glucose levels of VOCCH groups reduced from 29.8 (model control group) to 24.2, 22.4, 17.1, respectively, while VOCCA groups reduced from 32.2 (model control group) to 28.9, 28.1, and 25.9, respectively, which indicated that VOCCH would be better candidate as insulin enhancer than VOCCA.

As shown in Figure 9, the blood glucose concentrations of mice increased greatly after loading with D-glucose and then reduced smoothly. In the OGTT test, the blood glucose concentrations of normal mice remained less than 8 mM, while the model control group was consistently higher than that of the vanadium-treated groups. The blood glucose levels of diabetic mice remained above the basal levels at the end of the test. However, the blood glucose concentrations of vanadium treated groups were significantly lower than model control groups. Furthermore, the results of the OGTT indicated that impaired glucose tolerance was improved after treatment with VOCCH.

## 4. Discussion

Insulin-mimetic properties of vanadium salts and vanadium compounds have been widely reported in both type 1 and type 2 diabetic animal models [23–25]. Inorganic vanadium compounds have already been known to be effective in treatment of diabetic hyperglycemia, but the side effects, such as vomiting, diarrhea, hepatic, and renal toxicity limit their application [26, 27]. Organic vanadium coordination compounds have been proved with better absorption efficiency in gastrointestinal tract [28]. In this study, high doses of VOCCH and VOCCA exhibited antidiabetic activity at significantly lower intake levels of elemental vanadium compared to glibenclamide. Vomiting and diarrhea, the major side effects of vanadium compounds, were not observed. For example, BMOV and similar compounds like bis (ethylmaltolato) oxovanadium (IV) (BEOV) have already been in clinical trials [29]. However, the toxicity, lower bioavailability and nonlinear pharmacokinetics of these compounds compromise their pharmacological success [30]. These results indicated that the corporation of essential trace element vanadium and polysaccharide could enhance their biological activity, intercoordination, and bioavailability. The insulin-enhancing properties of organic vanadium complexes have previously been compared with those of inorganic vanadium salts [31, 32]. It was reported that vanadium-enriched* Cordyceps sinensis *was beneficial for contemporary treatment of depression and diabetes through the coeffect [33]. VOCCA and VOCCH also may be potential strategies for lowering the blood sugar level through the coeffect of vanadium and carboxymethyl-carrageenan and carboxymethyl-chitosan. In the present study, VOCCH, VOCCA, and VOSO_4_ showed similar hypoglycemic functions. However, the vanadium intake amount in the form of VOCCH and VOCCA was significantly lower than that of VOSO_4_ [22]. These results indicated that the anti-diabetic abilities of VOCCH and VOCCA were more effective than VOSO_4_.

## 5. Conclusion

A beneficial role of enhancing anti-diabetic actions of VOCCH and VOCCA was found in this study. VOCCH and VOCCA would be good candidates of insulin enhancers to effectively activate glucose metabolism *in vivo* and regulate glucose metabolism by influencing insulin levels. The ligands of carboxymethyl carrageenin and carboxymethyl chitosan allowed vanadium to supply at the minimal doses, prolonged the duration of drug activity, and reduced the toxicity of vanadium. This implied that carboxymethyl carrageenin and carboxymethyl chitosan would be promising biocompatible and biodegradable vehicles for the delivery of VO^2+^ ions.

Insulin receptor which is a kind of transmembrane glycoprotein complex molecules consists of two alpha subunits (135 kd) and two beta subunits (95 kd); they linked by three disulfide bonds. Alpha subunits are outside of cell; beta subunits are embedded in membranes. The combination of insulin and alpha subunits caused phosphorylation of multiple tyrosine residues of beta subunits, which activated protein tyrosine kinase (PTK) and triggered a series of cascade amplification reaction of phosphorylation and dephosphorylation to adjust the metabolism. PTPase (protein tyrosine phosphatase) is the main negative regulatory factor in insulin signaling pathways; it caused dephosphorylation of insulin receptor and its substrate and weakened insulin signals.

**Figure 10:**
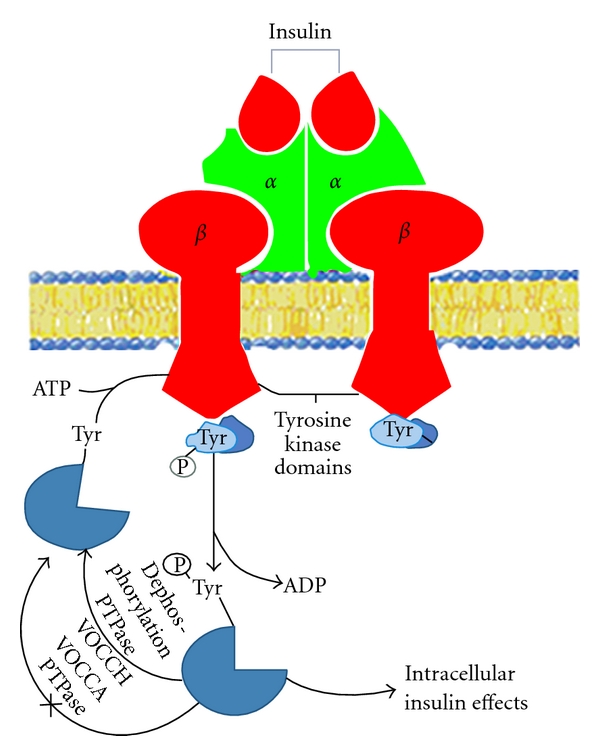
Schematic presentation of insulin signal transduction and possible action mechanisms of VOCCH and VOCCA.

Possible mechanisms for hypoglycemic properties of VOCCA and VOCCH are as follows: VOCCA and VOCCH could inhibit the dephosphorylation activity of PTPase, so dephosphorylation of tyrosine kinase and downstream signal substrate will be inhibited. In this way, the action time of insulin signal was prolonged, so VOCCA and VOCCH could lower the blood sugar level (Figure 10).

## Figures and Tables

**Table 1:** Impacts of intragastric VOCCA on blood glucose levels in both normal and alloxan-diabetic mice.

Group	Dose (g·Kg^−1^)	0 week	3 week
Normal control group	0	5.7 ± 0.6	6.0 ± 0.16
Model control group	0	17.8 ± 4.6	32.2 ± 3.3
Glibenclamide control group	0.2000	17.9 ± 4.3	26.7 ± 2.6
Low-dose group	0.6250	18.0 ± 4.3	28.9 ± 4.5^ad^
Middle-dose group	1.2500	17.8 ± 4.4	28.1 ± 5.0^ad^
High-dose group	2.500	17.6 ± 4.3	25.9 ± 4.7^ac^

^a^ *P*< 0.01, ^b^ *P*< 0.05, versus normal control group; ^c^ *P* < 0.01,  ^d^ *P* < 0.05, versus model control group.

**Table 2:** Impacts of intragastric VOCCH on blood glucose levels in both normal and alloxan-diabetic mice.

Group	Dose (g·Kg^−1^)	0 week	3 week
Normal control group	0	5.7 ± 0.6	5.1 ± 0.5
Model control group	0	17.0 ± 4.7	29.8 ± 5.4
Glibenclamide control group	0.200	17.1 ± 4.5	21.4 ± 4.5
Low-dose group	0.3125	17.6 ± 4.7	24.2 ± 3.4^ad^
Middle-dose group	0.6250	17.5 ± 4.4	22.4 ± 5.6^ac^
High-dose group	1.2500	17.2 ± 4.3	17.1 ± 6.9^acf^

^a^ *P* < 0.01, versus normal control group; ^c^ *P* < 0.01, ^d^ *P* < 0.05, versus model control group; ^f^ *P* < 0.01, versus glibenclamide control group.
